# Impact of Thyroid Dysfunction on Hair Disorders

**DOI:** 10.7759/cureus.43266

**Published:** 2023-08-10

**Authors:** Ramadan S Hussein, Tarek Atia, Salman Bin Dayel

**Affiliations:** 1 Department of Internal Medicine, Dermatology Unit, College of Medicine, Prince Sattam bin Abdulaziz University, Al-Kharj, SAU; 2 Department of Medical Laboratory Sciences, College of Applied Medical Sciences, Prince Sattam bin Abdulaziz University, Al-Kharj, SAU

**Keywords:** thyroid disorders, androgenetic alopecia, alopecia areata, telogen effluvium, hair loss

## Abstract

Hair loss is a problem for everyone, regardless of their age or sex. The three most prevalent types of hair loss, telogen effluvium, alopecia areata, and androgenetic alopecia, have been associated with a variety of risk factors. Strong evidence links thyroid hormones (THs) to hair loss. THs control the growth, differentiation, metabolism, and thermogenesis of body cells. The skin is a significant target organ for THs; however, the cellular and molecular causes of thyroid dysfunction-related skin diseases remain unknown. Hyperthyroidism, hypothyroidism, and drug-induced hypothyroidism can induce widespread hair shedding. Little information is available regarding the incidence and effects of thyroid dysfunction on hair problems. This study aimed to review the impact and prevalence of thyroid disorders on hair loss. The conclusions drawn from this study highlight the underestimated prevalence and impact of thyroid disorders on hair loss. The review of scientific articles, including original research, review articles, and a case report, provides a comprehensive understanding of the topic. This research adds to the existing literature by enhancing our understanding of the relationship between thyroid dysfunction and hair disorders. It contributes to the body of evidence by reviewing relevant studies and summarizing the impact of thyroid disorders on hair loss. The study also highlights the gaps in knowledge and the need for more research in this area to improve the diagnosis and management of hair disorders associated with thyroid dysfunction.

## Introduction and background

Thyroid hormones (THs) play a crucial role in regulating various cellular activities, including thermogenesis, proliferation, metabolism, and differentiation, during post-embryonic vertebrate development. Thyroxine (T4), the main product of the thyroid gland, acts as a prohormone that is converted to the active triiodothyronine (T3) hormone by peripheral deiodination enzymes. T3 exerts its effects on sensitive cells by binding to nuclear receptors known as thyroid hormone receptors (TRs). Through their DNA-binding activity, TRs function as transcription factors that control gene expression. Two types of TRs, TRα, and TRβ, encoded by different genes, have been identified, although their precise roles remain unclear [1].

Thyroid-related skin diseases involve complex cellular and molecular processes that are not fully understood. The skin is a significant target organ for THs. Conditions such as hyperthyroidism, hypothyroidism, and drug-induced hypothyroidism have been associated with widespread hair shedding. In approximately 50% of individuals with hyperthyroidism and 33% with hypothyroidism, hair loss is observed. Hypothyroidism is thought to impede the division of epidermal and skin appendage cells, leading to the catagen phase and a delay in telogen hair re-entry into the anagen phase in some individuals [2]. Hyperthyroidism, on the other hand, stimulates the production of reactive oxygen species (ROS) in untreated individuals, resulting in oxidative damage, peroxidation of biomembrane lipids, and increased free radical formation in mitochondria [3]. However, the exact mechanisms by which hyperthyroidism causes hair loss are not yet fully understood. It is worth noting that hair shedding may occur months before the manifestation of other symptoms, and replacement medication often halts hair loss, except in cases of long-term atrophic hair follicles (HFs) associated with hypothyroidism [2].

Despite the significance of thyroid disorders in relation to hair loss, the prevalence and impact of these disorders on hair loss are still underestimated. In this review, we followed the Preferred Reporting Items for Systematic Reviews and Meta-Analyses (PRISMA) guidelines and conducted a comprehensive analysis of scientific articles published between 2010 and 2022, using keywords such as "hair loss," "thyroid dysfunction," "hyperthyroidism," "hypothyroidism," and "anti-thyroid treatment" in databases such as PubMed and Google Scholar [4]. We carefully selected 11 relevant articles for this review, including six original articles, four review articles, and one case report. The chosen articles aim to provide a comprehensive understanding of the topic, combining primary research that presents new findings and data, review articles that summarize and analyze existing literature, and a case report that adds valuable clinical insights. By incorporating a diverse range of article types, this review seeks to present a well-rounded and informed analysis of the impact of thyroid disorders on hair loss, considering both recent research findings and the broader context provided by review articles.

## Review

HF structure and function

HFs are essential for hair production and undergo a cyclic growth and renewal process [5]. External and internal factors influence this cycle, with even minor environmental changes impacting the duration of each phase: anagen (growth), catagen (regression), and telogen (resting) [6]. Disorders like telogen effluvium (TE), androgenetic alopecia (AGA), and alopecia areata (AA) result from specific alterations in the hair cycle. The coordinated transformation of the sebaceous gland, perifollicular dermis, and subcutis, governed by an unknown mechanism called "the hair cycle clock," plays a role in these changes [5].

**Figure 1:**
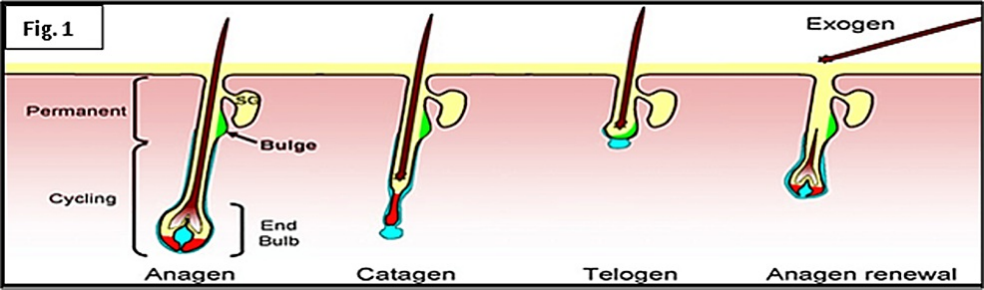
Structure of hair follicle

Adult HFs consist of mesenchymal dermal papilla (DP) cells and transient epithelial cells of the hair matrix, enclosing the outer and inner root sheath, hair shaft, and DP. Epithelial-mesenchymal coordination and bidirectional communication between pilosebaceous unit innervation and vasculature are crucial for cyclic HF development [7]. The upper and lower halves of the HF undergo significant fluctuations throughout the cycle, with the bulge zone (unilateral swelling of the outer root sheath) serving as an important anatomical landmark at the attachment of the arrector pili muscle (Figure 1) [8].

Stem cells (SCs) in adult HFs play a vital role in hair regeneration, tissue homeostasis, and epidermal repair. These slow-cycling, undifferentiated cells possess self-renewal and differentiation capabilities, supporting the rapid division of progenitor cells [9]. Various types of SCs, including mast cell precursors, melanocytic, mesenchymal, immature Langerhans, and neuronal SCs, have been identified [8]. The DP, located near the hair root, acts as a signaling center for hair growth, influencing the hair shaft's thickness, size, length, and diameter during the anagen phase [10]. HFs can autonomously grow when transplanted to another area of the skin and isolated human HFs exhibit biologically relevant properties in organ culture, including regulated cell growth, differentiation, death, and tissue rejuvenation [11].

Hair cycle

**Figure 2:**
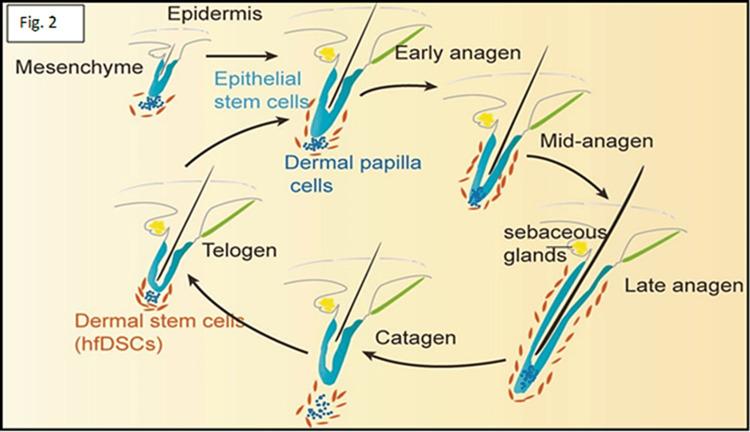
Hair cycle: functional HFs renew naturally through anagen, catagen, and telogen HF: Hair follicle

Traditionally, the hair cycle split into the growth (anagen I-VI), the regression (catagen), and the resting (telogen) phases (Figure 2). Normal scalp trichograms showed 86% anagen, 1% catagen, and 13% telogen hairs, with 100-150 hairs coming out daily [12]. The biological clock that ends anagen and begins catagen/telogen is intricate. Anagen lasts for 2-8 years, catagen for 4-6 weeks, and telogen for 2-3 months. Apoptosis of hair bulb keratinocytes begins with the catagen phase involution of the transitory follicle beneath the arrector pili muscle. This procedure takes two weeks to complete. The decreased follicle is dormant for two months until the following anagen cycle. The follicle sheds exogen hair late in the telogen phase, or early in the anagen phase.

Hair loss is now known as an exogen, whereas kenogen is a short period when the HF is empty [13]. Anagen stimulates matrix keratinocytes to develop inner root sheaths and hair fibers. It contains six morphologically distinct subphases, including pro-anagen and phases I-V. Subsequent meta-anagen causes the hair to develop on the epidermal surface. In the last phase of anagen, the follicle is deeply rooted in the subcutaneous tissue, whereas the bulb moves to a shallower location underneath the arrector pili muscle attachment. Involution of HF, apoptosis, and final differentiation conclude the anagen phase [14]. The catagen phase is the involution time. It is a two-to-four-week transitional period between anagen and telogen. The follicle underwent apoptosis-related morphological and molecular alterations during this period. Involution begins when bulb melanin synthesis ceases. Matrix melanocytes quit making melanin and absorb dendrites, while keratinocytes quit proliferating and terminally differentiate [6]. The DP-lateral stem cell population is protected from apoptosis during catagen, thereby enabling early anagen reproduction [10]. The follicle rests in the telogen phase after regression. The telogen lasts 3-4 months. The lower part of the hair loses its connection with the follicular sac and falls out. A recent study demonstrated that hair loss is governed by an active process, unlike telogen, which is inactive [15]. Although the exogen process is unknown, hair root architecture implies proteolytic processes between cells near the telogen hair base. Kenogen is the HF empty period between telogen hair loss and a new anagen hair outbreak. AGA worsening is characterized by increasing kenogen hairs and vellus hair and decreasing regular hair cycles [16].

Regulation of the hair cycle

Alterations in the hair cycle have clinical implications for hair growth. The regulation of skin appendage development involves a complex interplay of signaling chemicals, receptor expression changes, tissue biology, and epigenetic controls [17]. Recent theories suggest that changes in signal transmission between DP and bulge zone cells contribute to the cycling of the HF system, which has its own independent clock [18].

Autocrine, paracrine, and endocrine signaling systems govern HF differentiation, involving various cytokines, enzymes, neurotransmitters, hormones, and transcription factors. Signaling molecules such as the Wnt family, TGF-β, Hedgehog pathways, and FGF influence hair and appendage development [19]. The regeneration of HFs during the anagen phase is initiated by DP cells reaching the bulge region of epidermal stem cells. Pathways involving β-catenin, Wnt proteins, noggin, and Stat3 transcription factor are crucial for anagen induction [20]. Activation of Stat3 promotes HF development and biological processes, while cytokines/growth factors like EGF and IL-6 activate Stat3 signaling [21]. Wnt/β-catenin signaling is involved in mammalian hair development, and androgenic signaling in DP inhibits HF stem cell development in androgenetic alopecia [22]. Factors like HGF and SHH contribute to anagen growth and the transition of telogen follicles to the anagen phase [23]. VEGF, IGF-1, and TRH also play roles in extending the anagen phase duration [24,25].

TRH enhances hair shaft extension, lengthens the anagen phase, and prevents TGF-2 from terminating the phase. THs directly impact human HFs, affecting a variety of hair biology variables, from HF cycling to pigmentation. Scalp HFs serve as both the source and the target of the strong hair-growth stimulant TRH. During embryonic development, TH influences the differentiation and growth of HFs, ensuring their normal structure and function. THs promote the initiation and maintenance of the anagen phase by stimulating cell proliferation and metabolic activity within the HFs. They increase the rate of hair growth and contribute to the production of new hair fibers. THs help regulate the duration of the telogen phase by influencing the transition of HFs from the resting phase back to the growth phase. Moreover, THs play a role in synchronizing the shedding process, ensuring that HFs shed at appropriate intervals. THs also influences the pigmentation of hair. They stimulate the production and distribution of melanin, the pigment responsible for hair color [26].

Polyamine spermidine regulates the anagen phase and inhibits catagen, impacting cellular processes and stimulating hair development and epithelial stem cell biology [27,28]. Nestin, a marker for DP stem cells, plays a role in HF regeneration [29]. The transition from anagen to catagen is influenced by changes in hormone levels. Decreased levels of anagen-promoting hormones (IGF-1, HGF, and FGF-5S) and increased levels of inhibitors (TGF-β1, TGF-β2, and FGF) impact follicle development [30]. DKK-1 inhibits Wnt-mediated β-catenin signaling and promotes apoptosis of follicular keratinocytes during the transition [31]. TNF-related molecules and neurotrophins also contribute to the anagen-catagen regulation [32,33].

The mechanisms of the resting phase (telogen) are not fully understood, but telogen-arrested follicles contain 17-β estradiol and BMP4, which govern hair cell precursor survival and specification [18,34]. Cyclic epithelial FGF18 regulates the hair cycle during the non-growth period, influencing mitotic activity and follicular morphogenesis [35]. Msx-2 and protease cathepsin L regulate the exogen stage of the hair cycle [5]. Hormonal factors, including autocrine and paracrine hormones produced by balding DP cells influenced by DHT, may contribute to male pattern baldness. Upregulation of IL-6 inhibits hair shaft elongation, while 17β-estradiol has differential effects on hair growth depending on the scalp region [18,36].

Action and metabolism of THs

The hypothalamic-pituitary-thyroid (HPT) axis controls the amount of TH in the blood. Hypothalamic TRH promotes anterior pituitary gland thyroid-stimulating hormone (TSH) release. TSH activation in the thyroid produces T4, which has poor sensitivity for nuclear TRs, and T3, which is the main active metabolite. The biological function of TH is tightly controlled by TH transfer throughout the cellular membrane, an intracellular transformation of T4 into T3 or deactivation, the interaction of T3 with nuclear TRs, and DNA binding. These processes contribute to the regulation of the transcription of many TH target genes' transcription [37]. Besides nuclear TR mediation, TH signaling also involves cellular proteins such as membrane integrin αvβ3, which are considered TH non-genomic actions. Three selenocysteine-containing iodothyronine deiodinase enzymes (D1, D2, and D3) control target cell TH concentrations [38]. These enzymes metabolize TH by removing one iodine atom from the phenolic or tyrosyl rings of T4 and T3 via monodeiodination. Particularly, D1 and D2 remove iodine from the outer (phenolic) ring of T4 to convert T4 to T3 [39]. D3, TH's biological inhibitor, deiodinates the inner (tyrosyl) ring of T4 to inactivate T3 and create T2 or rT3 [40]. D1 and D3 are plasma membrane deiodinase proteins, whereas D2 is in the endoplasmic reticulum [37]. TRs communicate TH to the target cells. T3-TRs complexes bind to TH response elements (TREs) in the chromatin to control target gene expression. THRA and THRB encode the Thra and Thrb isoforms of TH receptors, respectively. Finally, intracellular TH activity requires TH transporters to move iodothyronines across the cell membrane. Three TH transporters, OATP1C1, MCT8, and MCT10, exhibit high sensitivity to iodothyronines [41].

Impact of THs on skin physiology

THs affect embryonic epidermal differentiation, hair growth, barrier development, tissue repair, epithelial cell growth, and skin protein gene expression [42]. THs help the epidermis build a barrier during embryonic development by enhancing cholesterol sulfate cycle enzyme activity and lamellar granule formation. Remarkably, human skin and HFs exhibit completely functional HPT axis proteins, particularly TSH and TRH receptors [43]. TSH and TRH skin receptors govern epidermal physiology by inducing skin-specific gene expression, explaining the relationship between altered TH levels and the most prevalent dermatological illnesses [42]. TRs mediate the effects of TH on the skin. TRα and TRβ, two skin tissue TR isoforms, positively and negatively regulate gene promoter transcription, respectively. Epidermal and dermal cells, as well as skin appendages, express TRs and both receptors, which may affect the production of certain keratins in cultured cells, either positively or negatively [43,44].

Impact of THs on hair

THs are required for HF growth and maintenance, implying that hair loss might be a sign of thyroid dysfunction [40]. The TH's effect on rat hair growth cycles was studied by Hale and Ebling [45]. They discovered that intraperitoneal T4 administration reduced both the telogen and anagen phases of the hair cycle. Despite the increased turnover, the net hair length did not vary from untreated animals. The length of time before hair regrows after epilation was reduced by around 10%. Inducing hypothyroidism using propylthiouracil in water lengthened hair regeneration by 20%. Thyrotoxicosis causes fine, silky hair. Diffuse non-scarring alopecia is also possible. In vitro studies have shown that thyrotoxicosis is associated with enhanced hair development. DNA flow cytometry of detached anagen hairs from thyrotoxic patients showed 30% more S and G2 + M cell cycle stages compared with euthyroid controls [46]. However, hair alterations in thyrotoxicosis vary depending on the topically applied THs. Topical T3 for one-two weeks raised counts of hair counts in mice and rats, whereas intraperitoneal T3 lowered hair counts in thyrotoxic animals [47]. In males with androgenic alopecia, a topical combination of growth hormone, thyroxine, and insulin boosted hair counts over six months [48]. Hypothyroidism causes slow-growing, coarse, dry, brittle hair. Loss of the outer third of the eyebrow and/or diffuse alopecia may also be observed. Hypothyroidism-associated alopecia might be caused by hormonal effects on the onset and duration of hair growth (Table 1) [49]. Patients with alopecia have 8-28% thyroid disease; however, chronic autoimmune thyroiditis has not been linked to it [50]. In one study, Kasumagić-Halilović found that alopecia patients had more antithyroid autoantibodies (25.7%) than healthy individuals (3.3%) [51].

**Table 1:** Impact of altered thyroid hormone on skin pathophysiology

Hypothyroidism	Thyrotoxicosis
1- Epidermal alterations:	Epidermal alterations:
Coarse, scaly, thin, skin	Thin, smooth skin
Hair and Nail alterations	2- Hair and Nail alterations
Alopecia, loss of lateral third of eyebrows	Alopecia
Coarse, dry, brittle hair	Fine hair
Coarse, brittle, dull, thin nails	Soft, shiny, friable nails (Plummer’s nails, onycholysis)
3-Sweat gland alterations	
Xerosis (dry skin), decreased sweating	
4-Dermal alterations	
Myxedema (non-pitting edema), carotenemia	
Edema (face, hands, eyelids), pallor	

The possible link between the thyroid profile and the most common types of alopecia

Telogen Effluvium (TE)

Mild changes in TH levels in the blood have a significant impact on the growth of human scalp HFs. The hormones T3 and T4 directly affect the follicles, making them particularly sensitive during the active growth phase (anagen). When someone has an underactive thyroid (hypothyroidism), they may experience hair loss (alopecia), along with prolonged shedding (telogen effluvium), as well as dry, brittle, and lackluster hair [52]. Surprisingly, even in cases of an overactive thyroid (hyperthyroidism), hair shedding can occur, and the tensile strength of the hair shafts is reduced [53,54]. This has been observed in rats, lambs, mice, and humans, confirming the role of THs in regulating hair development. In human scalp HFs, TH signaling can extend the anagen phase of hair growth, modify the expression of specific keratins, promote the proliferation of hair matrix cells, and delay the onset of programmed cell death (apoptosis) that leads to HF regression (catagen). While THs have limited influence on the creation of new hair shafts, T3 and T4 are responsible for increasing energy production in the mitochondria of human scalp HFs [55].

Alopecia Areata (AA)

The cause of AA is not fully understood, but it is believed to involve genetic susceptibility, autoimmune processes, and potential stress as contributing factors [56]. AA patients have a higher prevalence of autoimmune conditions, including Hashimoto's thyroiditis [57]. Studies have shown that autoimmune thyroid diseases are more common in people with AA, and they are more likely to have thyroid dysfunction, such as subclinical hyperthyroidism and hypothyroidism [58]. AA patients also exhibit a higher incidence of anti-thyroid antibodies compared to controls [59]. Certain human leukocyte antigens (HLA), specifically HLA-DQB1*03, are associated with both AA and antibodies-induced hypothyroidism [60]. Approximately 5% of AA patients have subclinical hypothyroidism related to Hashimoto's thyroiditis, and a significant proportion experience some form of thyroid dysfunction [61]. Thyroid involvement should be considered as a comorbidity in AA, and it is more prevalent in females [62].

Androgenic Alopecia

Female pattern hair loss (FPHL), also known as androgenic alopecia, is a common type of hair loss in adult women. It is characterized by progressive shrinking of HFs and a decrease in hair count, especially in the central, frontal, and parietal regions of the scalp. FPHL is the most frequent cause of hair loss in women, particularly in older age groups [63]. A study conducted on menopausal women aged 50 to 65 years found a prevalence of 52.2% for FPHL, suggesting its impact on quality of life during menopause [64]. The relationship between thyroid disorders and the increasing prevalence of FPHL in aging women is not fully understood. Hypothyroidism was observed in 31.25% of FPHL patients in a study, but there was no correlation between the severity of hair loss and the presence of thyroid disease [65]. Male pattern alopecia (MPA) is different from FPHL in terms of follicle shrinkage. Some theories propose a connection between MPA and metabolic syndrome, insulin resistance, and/or hypogonadism, but this link is not universally accepted [66,67]. Early-onset androgenetic alopecia has been associated with low testosterone and dehydroepiandrosterone levels, and testosterone replacement in hypogonadism may contribute to a decrease in antithyroid antibodies in these patients [67,68]. A small study conducted in 2021 on autoimmune hypothyroidism patients, suggested that male patients without baldness may have a better response to TH replacement therapy compared to those with MPA [68]. However, more evidence is needed to support this finding.

Hair Changes Associated With Thyroid Malignancies

While hair changes are not a typical symptom of thyroid cancer, there are a few potential ways in which hair may be affected. In some instances, individuals with thyroid malignancies may experience hair loss or thinning hair. This hair loss is not exclusive to the scalp and can affect other body hair as well. Thyroid cancer, particularly when accompanied by thyroid dysfunction, can lead to changes in the hair's texture. Patients might notice that their hair becomes dry, brittle, and prone to breakage [53]. In rare cases, individuals with thyroid malignancies may observe changes in their hair color. This can involve a darkening or lightening of the hair, which is attributed to disruptions in the production of melanin, the pigment responsible for hair color [54].

## Conclusions

Thyroid illnesses have several negative effects, including skin damage. Decades of the study have shown that TH signaling affects epidermal growth, homeostasis, and HFs. TH signal modification may change the pathophysiological mechanisms of the HF cycle; however, the findings remain inconclusive. Understanding how THs and their regulatory molecules govern skin biology in wellness and illness might result in the use of TH agonists or inhibitors to treat different cutaneous and hair problems.
